# Neural Residual Correction for 3D Tooth Point Cloud Canonicalization

**DOI:** 10.3390/jimaging12060243

**Published:** 2026-05-29

**Authors:** Chawalit Chanintonsongkhla, Varin Chouvatut, Chumphol Bunkhumpornpat, Pornpat Theerasopon

**Affiliations:** 1 Department of Computer Science, Faculty of Science, Chiang Mai University, Chiang Mai 50200, Thailand; chawalit.ch@up.ac.th (C.C.); chumphol.b@cmu.ac.th (C.B.); 2 Department of Preventive Dentistry, School of Dentistry, University of Phayao, Phayao 56000, Thailand; 3 Department of Orthodontics, School of Dentistry, University of Phayao, Phayao 56000, Thailand; pornpat.th@up.ac.th

**Keywords:** deep learning, artificial intelligence, tooth canonicalization, point cloud registration, dental 3D imaging, PointNet, iterative closest point, principal component analysis

## Abstract

**Background**: Statistical shape modeling and generative tooth synthesis require dental point clouds in canonical poses. This study compared canonicalization methods and proposed a hybrid pipeline pairing principal-axis alignment with a neural orientation guide and a trained residual correction. **Methods**: Seven classical, neural, and hybrid methods were evaluated on 9060 upper tooth point clouds across seven classes from 3DTeethSeg (891 patients, 1805 held out for validation) and 1465 external first molars from FDI16. Alignment was measured by Chamfer Distance to per-sample target poses (CD Target, validation only), Chamfer Distance to class templates (CD Template, both sets), and geodesic rotation error. **Results**: Neural-guided PCA selection with residual refinement (gPCA-rPointNet) reached the lowest CD Target (0.62 ± 2.43 × 10^−3^) and geodesic rotation error (3.3 ± 14.5 degrees), with 98.2% of predictions below 15 degrees. On the external set, the four PCA-based methods gave a lower CD Template than methods without geometric initialization. **Conclusions**: A neural orientation guide placed before principal-axis candidate selection resolved the PCA eigenvector sign ambiguity responsible for 180-degree failures on near-symmetric tooth crowns. Residual correction further reduced rotation error. The same pipeline produced consistent canonical poses for first molars on the external dataset, with validation on other tooth classes remaining limited.

## 1. Introduction

**Figure 1:**
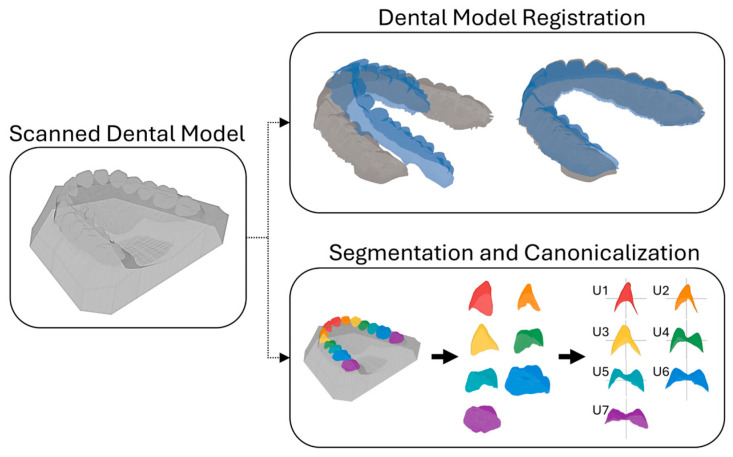
Examples of three-dimensional dental processing tasks. (**Upper**) Dental model registration aligns intraoral scans for prosthesis fabrication, implant planning, and orthodontic treatment assessment. (**Lower**) Segmentation into individual tooth instances and canonicalization into class-specific canonical poses with consistent anatomical axes. This study addresses the canonicalization task.

Intraoral scanners digitize the dentition as three-dimensional surface meshes, producing digital dental models that support clinical and research workflows spanning restoration design, orthodontic planning, and surgical simulation. Recent reviews have organized artificial intelligence applications in three-dimensional dental imaging into segmentation, registration, and soft-tissue prediction [1]. Generative deep learning methods have also been reviewed for automated dental restoration design, with architectures such as generative adversarial networks and transformers achieving error rates approximately half those of conventional digital methods [2]. Working with these models requires segmentation of individual tooth instances from full-arch scans, after which each isolated tooth can serve as input for shape analysis, statistical modeling, or generative synthesis. These downstream tasks operate on the assumption that teeth occupy consistent, standardized poses, because an arbitrary scan orientation carries no anatomical or geometric meaning shared across samples. Mapping each segmented tooth instance to a class-specific canonical pose through rigid transformation, a process referred to as canonicalization, provides this shared coordinate frame (Figure 1).

The dependency between pose standardization and shape analysis is well established. Mehl et al. aligned 170 lower first molars to a representative tooth, established dense point-to-point correspondence across the surfaces using an optical-flow-based algorithm, and applied principal component analysis (PCA) to the resulting shape vectors, finding that 20 components accounted for 83% of the total shape variance [3]. The alignment step was necessary because PCA identifies directions of maximum variance in the data. In a collection of arbitrarily oriented teeth, pose differences account for the largest share of total variance, and the leading principal components describe rotational offsets between samples rather than anatomical shape variation.

Generative deep learning methods require the same pose standardization but represent shape variation nonlinearly, encoding point clouds into compact latent spaces that can capture high-level shape features such as tooth roundness or tapered crown forms [4]. Within these learned representations, interpolation between latent codes produces smooth transitions between tooth shapes [4]. Linear PCA components, by contrast, follow orthogonal axes derived from the data covariance and may be insufficient for representing such nonlinear morphological features [4]. Ye et al. trained a variational autoencoder on 7732 pose-aligned upper first molar point clouds for generation, completion, and latent interpolation [5]. Chanintonsongkhla et al. trained continuous normalizing flow models on pose-aligned anterior tooth point clouds for shape generation and reconstruction from partial data [4], and a subsequent study reported cross-class latent translation from a single observed tooth through a shared latent space spanning seven upper arch classes [6]. Without pose alignment before training, learned latent spaces conflate shape variation with pose variation, making the resulting representations unreliable for downstream use.

Canonicalization can be approached at two levels. At the dataset level, an entire collection is aligned to a shared coordinate frame before downstream analysis. Large-scale shape repositories such as ShapeNet have employed PCA-based orientation with manual verification [7]. ConDor, a self-supervised method proposed by Sajnani et al., extended this line of work by learning canonical orientations directly from uncanonicalized collections without explicit pose annotations and producing canonicalized outputs for both full and partial point clouds [8]. In the dental domain, the FDI16 dataset provides 7732 pose-aligned maxillary first molars for shape generation and completion tasks [5]. The dataset’s x-axis points toward the adjacent tooth (FDI 17) and the y-axis along the occlusal direction, although the algorithmic procedure for deriving these axes was not detailed, and the dataset covers only a single tooth class. A multi-class dental canonicalization dataset spanning multiple tooth classes has not been established.

At the input level, individual instances are transformed into a canonical pose at inference time. In orthodontic treatment planning, per-tooth pose prediction has been used to arrange teeth into target occlusion. TANet combined PointNet features with graph-based spatial refinement to predict post-treatment transformations [9], and TAPoseNet used multi-scale graph convolution with explicit pose encoding for the same task [10]. Ding et al. formulated per-tooth orientation from intraoral scans as a six-degrees-of-freedom pose estimation task [11].

For isolated point clouds without full-arch context, canonicalization reduces to a registration problem, aligning each source to a class-specific template through rigid transformation. The Iterative Closest Point (ICP) algorithm is the dominant baseline for point cloud registration [12,13]. ICP iteratively minimizes the distance between corresponding points, converging monotonically to a local minimum, with subsequent work surveying ICP variants and efficiency improvements [14]. Principal component analysis (PCA) computes intrinsic shape axes via singular value decomposition of the point cloud covariance matrix [15], providing an orientation estimate independent of the input pose. A survey classified deep learning-based point cloud registration methods into supervised and unsupervised categories, covering correspondence-based, correspondence-free, and optimization-based approaches [16]. PointNet [17] introduced permutation-invariant global feature extraction from point clouds, and subsequent work extended this encoder to registration tasks. Aoki et al. proposed PointNetLK, applying the Lucas–Kanade algorithm in the PointNet feature space for iterative pairwise alignment [18]. Wang et al. proposed Deep Closest Point, using attention-based correspondence for learned registration [19]. Continuous 6D rotation representations [20] provide differentiable rotation prediction suitable for direct pose regression.

Unlike orthodontic arrangement, which predicts treatment-target positions from inter-tooth relationships, canonicalization maps individual teeth from arbitrary orientations to class-specific template poses for dataset preparation, particularly for generative modeling tasks that require pose-consistent training data. This study evaluated seven such methods, with the aim of producing a preprocessing pipeline that transfers to an independent external dataset for downstream generative applications. The seven methods span classical registration, neural, and hybrid pipelines. The contributions are as follows:

1. A multi-class canonicalization approach for seven upper tooth classes from arbitrarily oriented point clouds spanning the full SO(3) rotation space, combining neural orientation guidance with classical principal-axis alignment to resolve PCA eigenvector sign ambiguity on near-symmetric tooth crowns.

2. A systematic comparison of seven methods across classical, neural, and hybrid categories, with failure mode analysis and external validation on 1465 first molars from the external FDI16 dataset.

## 2. Methods

### 2.1. Datasets and Preprocessing

The training dataset was derived from the 3DTeethSeg challenge dataset [21]. Individual tooth meshes were extracted across all 32 FDI classes in both dental arches using Trimesh v4.9.0, producing approximately 24,000 tooth meshes. Each mesh surface was sampled to 15,000 points using Poisson disk sampling. The dataset was restricted to upper arch classes U1 through U7, representing FDI 11–17 and 21–27. Lower teeth and third molars were excluded. After filtering for sufficient crown structure and absence of surface artifacts, 9060 point clouds from 891 of the original 900 patients remained (Table 1) [6]. For each tooth class, a canonical template was selected from the 40 largest meshes by surface area. Template selection used ICP across a 7 × 7 grid of voxel sizes and maximum correspondence distances, retaining the template with the highest mean alignment fitness. Template selection was performed on the full dataset before splitting. Each remaining tooth was then ICP-aligned to its class template to establish a per-sample target pose, defined by registration to the template rather than by an independent anatomical reference. An 80/20 split at the patient level, stratified by tooth class, produced 7255 training samples and 1805 validation samples, with no tooth instance appearing in both partitions and no patient belonging to both sets [6].

A unified preprocessing pipeline normalized coordinates across all inputs before canonicalization. Each point cloud was centered at the origin by subtracting its centroid. A single global scale factor (*s* = 0.08076), computed as the reciprocal of the maximum bounding-sphere radius across all 9060 point clouds, was applied. Left-side teeth were mirrored across the sagittal plane by negating the x-coordinate, mapping them to their right-side equivalents. For each forward pass, 2048 points were randomly subsampled from the 15,000-point cloud to produce fixed-size inputs for neural methods. During training, a random rotation with axis uniformly sampled on the unit sphere was applied to each preprocessed point cloud, requiring methods to recover the canonical pose from arbitrary orientations spanning up to 180 degrees of geodesic distance.

Among the seven classes, first molars (U6) present a notable challenge for orientation disambiguation because their four-cusped rhomboidal crown outline produces similar point cloud geometry when rotated 180 degrees around the occluso-gingival axis, a distinction that typically requires dental expertise to resolve from shape alone. The validation partition of the FDI16 dataset [5] provided 1465 U6 first molar point clouds as an external test set. These scans were collected from patients undergoing aligner treatment using intraoral scanners. Because FDI16 lacks per-sample target poses, alignment quality on this dataset was measured exclusively against the canonical U6 template.

### 2.2. Iterative Closest Point with Principal-Axis Alignment

**Iterative Closest Point (ICP)** aligned each preprocessed source point cloud to its class-specific canonical template using the Open3D v0.19.0 point-to-point implementation [12,22]. Class-specific voxel sizes (0.125 to 0.50 mm) and maximum correspondence distance thresholds (2.0 to 8.0 mm) were set to the best-performing values identified during the ICP grid search used for template selection. Point pairs beyond the correspondence distance threshold were excluded from matching. All ICP parameters were defined in the original coordinate space and scaled by the global normalization factor for use in normalized coordinates. The maximum iteration count was set to 100.

**Figure 2:**
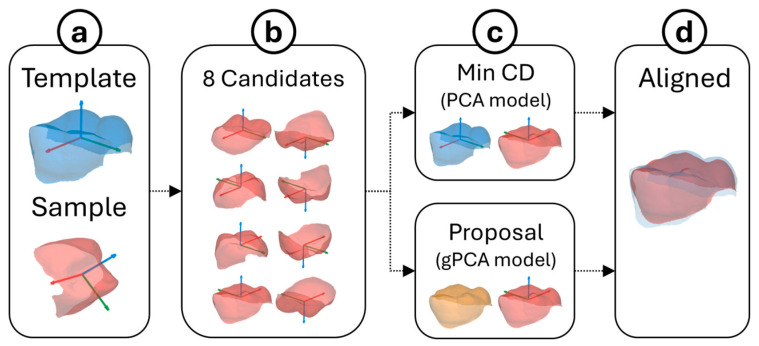
PCA versus gPCA axis–sign selection for a representative U6 first molar. (**a**) Sample and template with principal axes. (**b**) Eight PCA axis–sign candidates with corresponding principal-axis orientations. (**c**) PCA selects the candidate with the minimum Chamfer Distance (CD) to the template. gPCA uses a PointNet coarse alignment to establish approximate orientation, then selects the PCA candidate nearest to this proposal by geodesic rotation distance. (**d**) Final aligned sample overlaid on the canonical template.

**Principal-axis alignment with ICP refinement (denoted as PCA)** performed a two-stage alignment (Figure 2). In the first stage, singular value decomposition (SVD) computed principal axes for both source and template point clouds. Because PCA eigenvectors are defined only up to sign, each of the three axes can be independently negated, producing eight possible orientation candidates. All eight axis–sign combinations were evaluated, and the combination minimizing Chamfer Distance to the template was selected. In the second stage, ICP refined this coarse alignment using the same parameters as standalone ICP.

### 2.3. Neural and Hybrid Alignment

Five methods using neural components were evaluated. Two standalone neural methods and three hybrid pipelines combining classical and neural components are summarized in Table 2. PointNet was selected as the encoder backbone for its well-characterized global feature extraction, facilitating interpretable analysis of each pipeline stage. PointNetLK was included as a neural registration baseline using the original implementation [18]. Although more recent 3D encoders exist, the focus of this study was on pipeline design rather than encoder capacity.

**PointNet direct pose regression** used a PointNet encoder [17] to predict rigid transformations from preprocessed point clouds. The architecture comprised spatial transformer networks (STN3d, STN64), PointNet feature extraction producing 1024-dimensional global features, a 64-dimensional class embedding, and regression heads outputting a 6D rotation vector and 3D translation vector. The 6D rotation representation [20] was converted to a 3 × 3 rotation matrix via Gram–Schmidt orthogonalization. The model contained approximately 3.5 million parameters.

**PointNetLK** [18] used the Lucas–Kanade algorithm in the PointNet feature space to iteratively align source to template. A PointNet feature extractor (without spatial transformer networks) encoded both point clouds into 1024-dimensional global features. The Jacobian of the feature function with respect to SE(3) perturbations was approximated by finite differences in the template and precomputed once per forward pass. Every 10 iterative steps, the transformed source was re-encoded, the feature residual between source and template was computed, and a 6-dimensional twist update was solved via the pseudo-inverse of the Jacobian. The twist was mapped to a 4 × 4 rigid transformation via the SE(3) exponential map, and the cumulative transform was updated by left-multiplication. The model contained approximately 152,000 parameters and used the class template at inference.

**PCA followed by PointNet (rPointNet)** applied PCA pre-alignment before neural prediction. The PointNet component had identical architecture to the standalone model but received PCA pre-aligned inputs rather than arbitrarily oriented point clouds. The neural network learned only the residual correction between PCA output and the canonical pose. The model contained approximately 3.5 million parameters.

**PCA with PointNet guidance (gPCA)** addressed the PCA sign ambiguity by using PointNet to guide PCA candidate selection (Figure 2). In the first stage, the pre-trained PointNet predicted a coarse canonical alignment from the arbitrarily oriented input. In the second stage, PCA decomposition of the PointNet-aligned output produced eight axis–sign candidates. Rather than selecting the candidate with the lowest Chamfer Distance to the template, this method selected the PCA candidate whose rotation had the smallest geodesic distance to the PointNet-predicted coarse orientation. ICP then refined the selected candidate. This two-stage method used only the pre-trained PointNet, with no additional training.

**PCA with PointNet guidance and residual refinement (gPCA-rPointNet)** added a trainable PointNet that learned residual corrections from the gPCA output. The refinement PointNet had identical architecture to the coarse-alignment PointNet and used pre-trained weights via transfer learning, contributing 3.5 million additional trainable parameters on top of the 3.5 million frozen coarse-stage parameters. All three components ran sequentially during each training step. The frozen coarse PointNet processed the input without gradient computation, gPCA produced the PCA-refined alignment, and the trainable refinement PointNet predicted a residual correction with gradient flow.

PointNet, rPointNet, and gPCA-rPointNet were trained to regress a 6D rotation vector [20] and a 3D translation vector for each input. These predictions were converted to a 4 × 4 rigid transformation matrix. For each training sample, a random SO(3) rotation was applied to the target point cloud, and the composition of the predicted transform with the inverse target transform was evaluated against the identity matrix. PointNet, rPointNet, and gPCA-rPointNet used the mean squared error of this composition, while PointNetLK used its Frobenius norm [18]. Chamfer Distance was computed for monitoring but not used as a training objective for any method.

All neural and hybrid methods were implemented in PyTorch v2.8.0. Training used the AdamW optimizer [23] with an initial learning rate of 0.001 and weight decay of 0.0001. A step-based plateau scheduler reduced the learning rate by a factor of 0.5 after 3000 steps without improvement, with a minimum learning rate of 0.0001. Gradient norms were clipped to 1.0. Training ran for 50,000 steps with a batch size of 32 on a single NVIDIA RTX 3090 (NVIDIA Corporation, Santa Clara, CA, USA) graphics processing unit (Supplementary Figure S1) with a fixed random seed for reproducibility.

### 2.4. Evaluation Metrics

Chamfer Distance (CD) [24] quantified alignment quality as the sum of the two bidirectional mean-squared nearest-neighbor distances between two point clouds. All CD values are reported in normalized coordinate space after applying the global scale factor (*s* = 0.08076) and expressed as ×10^−3^. Two variants were used. CD Target compared the aligned point cloud to the per-sample target pose, available for the validation set where target poses were established. CD Template compared the aligned point cloud to the canonical class template, applicable to both datasets. For the external FDI16 dataset, where per-sample target poses were not available, only CD Template was computed. CD Template does not directly measure rotation error but serves as an indirect indicator of alignment quality. Low CD Template values generally correspond to well-aligned samples with morphology similar to the class template, while high CD Template values may reflect alignment failure, morphological difference from the template, or reduced surface overlap between the sample and the template.

Rotation error was the geodesic angular deviation (0 to 180 degrees) between predicted and target orientations, equivalent to the geodesic error metric used by Ding et al. [11], with per-axis components around the mesio-distal (X), bucco-lingual (Y), and occluso-gingival (Z) anatomical axes. Translation error was the distance between predicted and target tooth centroid positions in the canonical coordinate frame. Rotation and translation errors were computed only for the validation set, where target transformation matrices were available.

Summary statistics are reported as mean ± standard deviation. For overall (All-class) comparisons across six methods, Shapiro–Wilk tests on per-sample differences confirmed non-normal distributions, so pairwise Wilcoxon signed-rank tests with Bonferroni correction were used for method comparisons, with statistical significance assessed at the α = 0.05 level.

## 3. Results

Seven canonicalization methods were evaluated on the validation set (*n* = 1805) and the external FDI16 dataset (*n* = 1465). Single-step approaches included Iterative Closest Point (ICP), principal component analysis (PCA), PointNet, and PointNetLK. Hybrid pipelines combined PCA with neural components and included residual PointNet correction (rPointNet), neural-guided PCA selection (gPCA), and gPCA with residual refinement (gPCA-rPointNet). Mean values with bootstrap 95% confidence intervals for rotation error, translation distance, and Chamfer Distance across all methods and tooth classes are consolidated in Supplementary Table S1.

### 3.1. Training Convergence

The four trainable methods (PointNet, PointNetLK, rPointNet, gPCA-rPointNet) converged within 50,000 training steps (Supplementary Figure S1). PointNetLK’s training loss converged, but its predicted transformations did not establish the per-sample target pose.

### 3.2. Rotation Error

Geodesic rotation error quantified the angular difference between predicted and target orientations (Table 3). PointNetLK produced 114.8 ± 45.8 degrees, indicating that its linearized inverse-compositional update did not establish the canonical pose under full SO(3) augmentation, and was excluded from the per-class table. The occluso-gingival axis contributed the largest per-axis error across all methods (Supplementary Figure S2).

Among single-step methods, ICP achieved a mean rotation error of 20.4 ± 33.1 degrees, with low error for most samples but scattered high-error failures. PCA achieved 32.4 ± 64.2 degrees overall, with a bimodal distribution in which 65.2% (1177/1805) of samples fell below 5 degrees, but 16.0% (289/1805) exceeded 150 degrees, clustering near 180 degrees predominantly around the occluso-gingival axis (Supplementary Figure S2). PointNet achieved 13.5 ± 19.7 degrees with a unimodal distribution and no 180-degree flip cluster.

Among hybrid methods, rPointNet rotation error (11.4 ± 37.8 degrees) remained bimodal, with 4.8% (86/1805) of samples exceeding 150 degrees. gPCA produced a unimodal distribution with a mean rotation error of 5.9 ± 19.3 degrees. gPCA-rPointNet achieved the lowest rotation error (3.3 ± 14.5 degrees) with per-axis errors of 1.2 degrees (mesio-distal), 1.1 degrees (bucco-lingual), and 2.3 degrees (occluso-gingival). Of gPCA-rPointNet predictions, 93.2% (1682/1805) fell below 5 degrees, 97.5% (1759/1805) below 10 degrees, and 98.2% (1773/1805) below 15 degrees.

### 3.3. Alignment Quality by Chamfer Distance

CD Target measured alignment to per-sample target poses on the validation set (*n* = 1805). The four PCA-based methods clustered at the low end, with gPCA-rPointNet at 0.62 ± 2.43, gPCA at 0.74 ± 2.68, PCA at 1.60 ± 2.42, and rPointNet at 1.71 ± 3.65. PointNet alone reached 2.66 ± 2.97, ICP reached 3.05 ± 6.86, and PointNetLK collapsed at 131.35 ± 273.35, comparable in magnitude to random-rotation predictions.

CD Template measured alignment to the canonical class template on the same validation set. Per-class CD Template is reported in Table 4. At the All-class level, the four PCA-based methods formed a tight cluster, with gPCA at 7.39 ± 4.91, gPCA-rPointNet at 7.57 ± 5.27, PCA at 7.63 ± 4.93, and rPointNet at 8.24 ± 5.54, a within-group spread of approximately 0.85 × 10^−3^ from best to worst. PointNet alone reached 8.50 ± 4.69, ICP reached 10.23 ± 7.19, and PointNetLK reached 121.44 ± 238.45 and was excluded from the per-class table.

**Figure 3:**
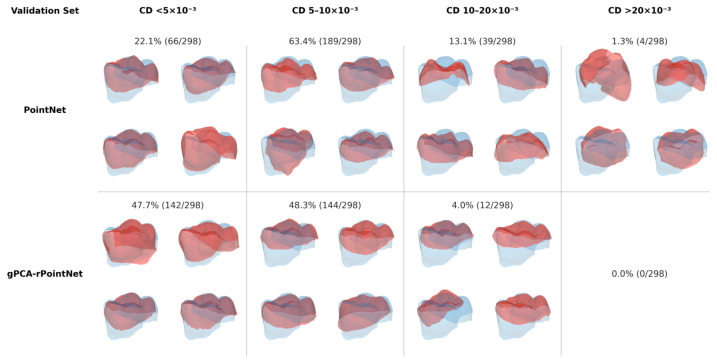
Alignment quality for U6 first molars on the training validation set (*n* = 1805) by CD Template bin (×10^−3^: <5, 5–10, 10–20, >20). PointNet and gPCA-rPointNet are shown, and the full six-method comparison is in Supplementary Figure S3. Each cell is a representative aligned sample (red) overlaid on the canonical template (blue).

Alignment quality varied across tooth classes (Table 4). Among the four PCA-based methods, U1 central incisors produced the highest CD Template, while PointNet’s highest CD Template was on U5 second premolars and ICP’s on U6 first molars. Representative alignment quality across CD Template bins for PointNet and gPCA-rPointNet is shown in Figure 3, with the full six-method comparison in Supplementary Figure S3.

**Figure 4:**
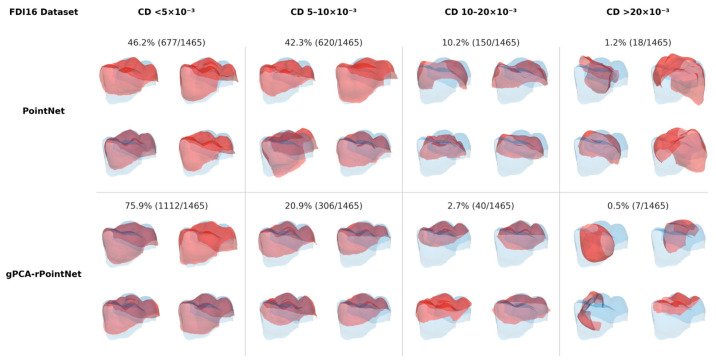
Alignment quality for external FDI16 first molars (*n* = 1465) by CD Template bin, with the same bins and rendering as Figure 3. PointNet and gPCA-rPointNet are shown, and the full six-method comparison is in Supplementary Figure S4.

On the external FDI16 dataset of 1465 first molars, the four PCA-based methods produced a consistent CD Template, with gPCA at 4.09 ± 3.27, PCA at 4.11 ± 3.12, rPointNet at 4.21 ± 3.22, and gPCA-rPointNet at 4.22 ± 4.03. PointNet alone reached 6.39 ± 3.89, ICP reached 26.96 ± 37.56, and PointNetLK reached 178.77 ± 561.31. Rotation and translation errors were not computed for FDI16 because per-sample target transformations were not available. Of gPCA-rPointNet samples, 75.9% (1112/1465) fell below CD Template 5.0, with seven samples (0.48%) exceeding 20.0 (Figure 4).

## 4. Discussion

Mapping three-dimensional shapes into a standardized coordinate frame is a prerequisite for geometric analysis at any scale, from pairwise specimen comparison to dataset-wide shape decomposition. At the specimen level, template-based registration establishes point correspondences between a source and a reference. Template-based registration pipelines combining rigid and deformable stages have been applied to skeletal morphometry across diverse taxa [25]. Nagawa et al. applied ALPACA deformable registration to femoral bones before Generalized Procrustes Analysis and principal component analysis (PCA), identifying shape modes associated with knee osteoarthritis severity [26]. At the dataset level, the ShapeNetCore project aligned approximately 51,300 models across 55 object categories through PCA-based candidate orientations with human expert verification [7]. Whether the task is pairwise registration or dataset-level canonicalization, the underlying geometric problem is the same. Both reduce to recovering the rigid transformation that maps an arbitrary source pose to a shared reference frame.

In digital dentistry, deep learning has advanced segmentation, registration, and soft-tissue prediction [1], with generative methods reviewed separately for automated tooth reconstruction [2]. Both statistical shape decomposition [3] and generative latent-space models [4,6] depend on pose-aligned input data. However, existing dental datasets either cover a single tooth class with undocumented orientation axes [5] or provide no per-tooth canonical poses [21]. The canonicalization pipeline evaluated in this study addresses this gap by producing multi-class canonical poses from arbitrarily oriented inputs, with the resulting poses already serving as inputs for cross-class tooth shape generation [6]. The seven methods evaluated in this study span classical, neural, and hybrid strategies to address these specific task challenges.

Recovering a rigid transformation from a tooth point cloud to a class template can be approached in two ways. Classical registration iteratively minimizes a geometric cost function between the source point cloud and the template, with Iterative Closest Point [12,13] minimizing the sum of squared point-to-point distances at each step and converging to a local minimum. Principal component analysis (PCA) pre-alignment provides an initial orientation by orienting the source along its principal axes [15], reducing ICP’s sensitivity to the starting pose. Deep learning methods share a common pattern. A learned network extracts permutation-invariant feature descriptors from each input point cloud, and a downstream head converts those descriptors into a rigid transformation [16]. Approaches diverge at the downstream head. Correspondence-based methods match descriptors between source and template and solve for the transformation in closed form, while correspondence-free methods regress the transformation directly from the pooled global features, either in one forward pass or iteratively in feature space [16]. Both branches benefit from continuous rotation parametrizations such as the 6D representation of Zhou et al., which removes the discontinuities of quaternion or Euler encodings and stabilizes gradient-based training under arbitrary initial orientations [20].

In this study, alignment quality was quantified by two metrics. Geodesic rotation error measured the angular deviation between the predicted and target orientations, providing a direct measure of pose correctness, equivalent to the metric used by Ding et al. for tooth pose estimation in intraoral scans [11]. Chamfer Distance (CD) measured similarity between two point clouds as the sum of bidirectional mean-squared nearest-neighbor distances and is widely used as a quantitative metric in 3D point cloud generative work [27,28]. Two CD variants were reported. CD Target compared each aligned point cloud to its per-sample target pose, available only on the validation set. CD Template compared each aligned point cloud to the canonical class template, applicable to both the validation set and the external set. CD Template is dominated by bidirectional point cloud overlap with the template and is insensitive to small rotational misalignments, especially for teeth whose overall shape looks similar after a 180-degree flip around a principal axis (see Figure 2), so it does not reflect rotation correctness as directly as the rotation error metric does.

The two classical methods displayed different failure modes. ICP alone produced a mean rotation error of 20.4 ± 33.1 degrees with a distributed pattern of high-error samples scattered across the validation set, consistent with its known sensitivity to local minima in the cost function when the initial pose is far from optimal [12,14]. PCA produced a bimodal rotation error distribution, with 65.2% (1177/1805) of samples falling below 5 degrees and 16.0% (289/1805) exceeding 150 degrees. Principal-axis decomposition is geometrically determined only up to independent flipping of each eigenvector, so for shapes with near-bilateral symmetry, multiple axis–sign assignments fit the same point cloud equally well. Teeth show such approximate symmetry, particularly molars, where buccal and lingual surfaces are difficult to distinguish from point cloud geometry alone without anatomical knowledge. Correctly and incorrectly oriented PCA candidates were close in CD cost, and ICP refinement could not recover from a misoriented start. Improving PCA required better candidate selection at the decomposition step rather than finer alignment afterward. The cost function alone could not distinguish correct from incorrect candidates.

Direct PointNet regression produced a unimodal rotation error distribution at 13.5 ± 19.7 degrees, with 1.0% (18/1805) of samples exceeding 150 degrees. Unlike ICP or PCA, PointNet produced its estimate from a single forward pass without iterative geometric refinement, and on inputs that were already close to aligned, this non-iterative estimate did not match the precision the classical methods reached through their refinement loops. Two variants extended this baseline. The first, rPointNet, placed a trained PointNet residual correction after PCA pre-alignment, reducing the mean rotation error from PCA’s 32.4 ± 64.2 degrees to 11.4 ± 37.8 degrees, but did not eliminate the 180-degree cluster, and 4.8% (86/1805) of samples still exceeded 150 degrees. A neural component placed after PCA (rPointNet) can refine the continuous residual rotation but cannot flip the discrete sign error of the prior, so the 180-degree failure mode persists and residual correction cannot resolve it. Distinguishing the correct orientation from a 180-degree flip on a near-symmetric molar typically requires expertise in dental anatomy, and a non-expert observer can struggle to make this distinction from shape alone (Figure 2).

In gPCA, the neural prediction ran before the PCA decomposition. The same PointNet that served as a direct regressor produced a coarse orientation prediction sufficient to disambiguate among the eight PCA axis–sign candidates. Although feature-learned regression is less geometrically precise than principal-axis decomposition, it is less prone to the 180-degree failures that PCA’s eigenvector sign ambiguity introduces (Table 4), making PointNet’s prediction usable as a sign-disambiguation prior despite lower angular precision. Selecting the PCA candidate geodesically nearest to the PointNet proposal ensured commitment to the correct octant, after which ICP refined the geometry. The resulting mean rotation error of 5.9 ± 19.3 degrees combined PointNet’s robustness to arbitrary orientations with PCA’s geometric precision, without requiring any additional training.

Adding a trained residual PointNet on top of gPCA (gPCA-rPointNet) further reduced mean rotation error to 3.3 ± 14.5 degrees, reaching a more refined pose than gPCA’s template-matching alone could achieve. gPCA-rPointNet placed a neural orientation guide before classical principal-axis alignment to resolve the discrete sign ambiguity, then placed a trained correction after to refine the continuous residual. On the internal validation set, where per-sample CD Target and geodesic rotation error are available, the combined pipeline produced the lowest mean CD Target and rotation error among the six methods evaluated (Supplementary Table S1). On CD Template, gPCA-rPointNet was comparable to the other PCA-based methods, as this metric is a coarse, template-based measure of overall shape match rather than of the fine rotation that separates the methods. However, gPCA-rPointNet had the highest inference time (Table 2). The standard ICP registration steps account for most of this runtime, while its neural components are inexpensive. As a one-time preprocessing step, this added cost is negligible in practice, given the lower rotation error and the elimination of PCA sign-flip failures.

Alignment quality varied across tooth classes (Table 4), consistent with differences in crown geometry and symmetry. Upper central incisors U1, with planar crown surfaces, produced the highest CD Template for the four PCA-based methods, while PointNet produced its highest CD Template on U5 second premolars and ICP on U6 first molars. Multi-cusped molars U6 and U7 showed the widest method-dependent variation, in line with both the cusp-arrangement complexity of multi-cusped crowns and the per-sample variability of scanned surface detail across individual tooth instances. PCA pre-alignment resolved the competing local minima that these geometry variations can produce.

Published work on neural tooth pose estimation for individually segmented point clouds remains sparse, and the closest comparable study is Ding et al., who proposed a deep learning model (TP-Net) for tooth pose estimation on 3D intraoral scans, formulated by predicting the rigid transformation from each tooth’s scan-acquired orientation to a local anatomical coordinate frame [11]. TP-Net was evaluated against four standard point cloud encoders (PointNet, PointNet++, PointCNN, and MinkCNN) and reached a mean geodesic rotation error of 6.86 degrees on their held-out test set, compared to 12 to 15 degrees for the encoder baselines. The starting pose in that task is the orientation at which each tooth was acquired by the intraoral scanner, which is typically close to the anatomical frame and therefore requires only a small corrective rotation. The canonicalization task in this study is harder in that each tooth is rotated uniformly across the full SO(3) range during training augmentation, removing any prior alignment to the anatomical coordinate frame. Under this condition, gPCA-rPointNet reached a mean geodesic rotation error of 3.3 ± 14.5 degrees. Ding et al. noted that 5 to 7 degrees is generally acceptable for orthodontic applications, as even human expert annotations show approximately 5 degrees of inconsistency for the same tooth [11].

On the external FDI16 dataset, the four PCA-based methods produced consistently oriented alignments across the 1465 first molars, with CD Template comparable between the training and validation U6 samples and the external set (Supplementary Figure S4). Across the four CD Template bins, the aligned first molars showed visually consistent poses on the external set, matching the pattern seen on the training validation samples. A small number of outliers remained, with 7 of the 1465 (0.48%) exceeding CD Template 20.0, where the aligned orientation still tracks the canonical template but the crown morphology and scanned surface detail differ from the typical first molar shape (Figure 4). Per-sample target rotations were not available for this external set, but the visually consistent pose pattern across the full CD Template distribution was sufficient to support downstream uses such as producing pose-aligned generative inputs [6]. Generalization across all seven tooth classes on external data remains to be established.

PointNetLK was included as a representative learning-based registration baseline that operates iteratively in PointNet feature space [18]. The original method was trained and evaluated with rotation perturbations of 0 to 45 degrees, consistent with its inverse-compositional Lucas–Kanade formulation around a small initial guess. Applied to the full SO(3) range used in this study, PointNetLK did not reproduce reliable alignments, suggesting that the linearization assumption of its inverse-compositional formulation holds mainly within the method’s designed operating range. Although PointNetLK in this configuration was not well-suited to arbitrary-pose canonicalization, this reflects its use outside the designed operating range and should not be taken as representative of modern deep registration methods more broadly. Comparison against recent stronger correspondence-based or transformer-based registration methods can be explored in future work.

## 5. Limitations

Several limitations remain in this study. A single canonical template per class was selected from the full dataset before the training–validation split, which introduces some validation dependence because the same reference frame is shared. Selecting each class template from the training partition would strengthen the validation. An independent landmark-based reference could also offer an alternative to constructing the reference frame from a single template.

The canonicalization pipeline operates on individually segmented tooth instances and does not use full-arch context. Relative positions of adjacent teeth on the dental arch could provide additional spatial cues that help disambiguate orientation, but incorporating arch-level information would require architectural extensions such as multi-tooth spatial encoding or a consensus mechanism across neighboring teeth, which would increase model complexity.

External validation covered U6 first molars only, because no other public dental dataset with pre-aligned per-tooth instances was available at the time of this study. On this set, per-sample target poses were absent, so alignment could be assessed only by template overlap, an indirect measure that does not capture rotation accuracy. Extending the evaluation to the remaining upper tooth classes, and to datasets that permit per-sample rotation assessment, is a direction for future work.

## 6. Conclusions

A neural orientation guide placed before classical principal-axis candidate selection resolved the eigenvector sign ambiguity that produces 180-degree failures on near-symmetric tooth crowns. A trained residual correction further reduced the remaining rotation error. The same pipeline also produced consistent canonical poses for first molars from an external dataset, with validation on other tooth classes remaining limited.

## Figures and Tables

**Table 1:** Dataset composition by tooth class.

Tooth Class	FDI Notation	N (Training)	N (Validation)	N (Total)
Training Dataset				
U1 (Central Incisor)	11, 21	1152	287	1439
U2 (Lateral Incisor)	12, 22	1167	291	1458
U3 (Canine)	13, 23	937	233	1170
U4 (First Premolar)	14, 24	1137	283	1420
U5 (Second Premolar)	15, 25	1071	266	1337
U6 (First Molar)	16, 26	1198	298	1496
U7 (Second Molar)	17, 27	593	147	740
Total		7255	1805	9060
FDI16 Dataset				
U6 (First Molar)	16	-	1465	1465

**Table 2:** Model architecture, parameter count, end-to-end inference time per 1000 samples, and a brief description. Frozen parameters are inherited from the pre-trained PointNet model.

Method	Parameters	Inference (1000 Samples)	Description
ICP	-	15.9 s	Point-to-point ICP to class template
PCA	-	54.3 s	PCA pre-alignment (8 candidates) + ICP refinement
PointNet	3.5 M	7.2 s	Direct 6D rotation + translation regression
PointNetLK	152 K	71.8 s	Lucas–Kanade in PointNet feature space
rPointNet	3.5 M	68.9 s	PCA pre-alignment then PointNet residual correction
gPCA	3.5 M	68.9 s	Frozen PointNet guides PCA sign selection then ICP
gPCA-rPointNet	7.0 M	97.8 s	gPCA (3.5 M frozen) then trained PointNet residual

**Table 3:** Rotation error by method on the training validation set (*n* = 1805), reported as per-class mean ± SD in degrees and as the percentage of samples at or below each geodesic rotation threshold.

Tooth Class	ICP	PCA	PointNet	rPointNet	gPCA	gPCA-rPointNet
Geodesic rotation error by tooth class (degrees)						
All	20.4 ± 33.1 ^d^	32.4 ± 64.2 ^d^	13.5 ± 19.7 ^c^	11.4 ± 37.8 ^b^	5.9 ± 19.3 ^b^	**3.3 ± 14.5** ^a^
U1	3.8 ± 14.6	45.1 ± 73.1	8.3 ± 5.8	5.7 ± 23.0	5.7 ± 16.2	**2.0 ± 3.9**
U2	**3.1 ± 5.4**	63.1 ± 79.7	12.0 ± 18.4	9.6 ± 27.8	12.5 ± 30.8	5.1 ± 18.6
U3	3.5 ± 11.8	21.7 ± 48.0	9.7 ± 7.3	9.2 ± 31.4	6.4 ± 12.3	**3.2 ± 11.6**
U4	9.3 ± 23.7	3.9 ± 21.1	10.4 ± 10.8	2.4 ± 15.1	**1.6 ± 1.2**	1.8 ± 10.4
U5	14.6 ± 32.6	46.5 ± 73.2	14.8 ± 21.8	17.2 ± 46.5	7.3 ± 18.5	**6.2 ± 23.8**
U6	62.6 ± 22.9	18.0 ± 52.3	16.5 ± 22.5	18.3 ± 52.2	2.7 ± 17.5	**1.8 ± 9.6**
U7	60.6 ± 40.3	22.3 ± 55.9	30.2 ± 38.4	22.5 ± 55.6	4.9 ± 23.3	**3.5 ± 14.1**
Rotation error percentiles across all classes (%)						
≤5°	62.4%	65.2%	14.9%	82.0%	77.5%	**93.2%**
≤10°	71.7%	75.0%	55.5%	92.3%	92.0%	**97.5%**
≤15°	73.9%	78.4%	80.3%	93.6%	96.0%	**98.2%**
≤20°	75.0%	80.5%	89.4%	94.3%	97.0%	**98.6%**
>150°	0.6%	16.0%	1.0%	4.8%	0.8%	0.7%

Methods sharing the same superscript letter in the All row are not significantly different (Wilcoxon signed-rank, Bonferroni-corrected *p* > 0.05).

**Table 4:** Chamfer Distance to canonical template (CD Template, × 10^−3^) by tooth class on the training validation set (*n* = 1805) and the external FDI16 dataset (*n* = 1465). Values are mean ± SD.

Tooth Class	ICP	PCA	PointNet	rPointNet	gPCA	gPCA-rPointNet
Training Dataset						
All	10.23 ± 7.19 ^d^	7.63 ± 4.93 ^c^	8.50 ± 4.69 ^e^	8.24 ± 5.54 ^d^	**7.39 ± 4.91 ^a^**	7.57 ± 5.27 ^b^
U1	13.10 ± 8.44	12.59 ± 5.70	**11.57 ± 3.60**	13.76 ± 6.26	12.19 ± 6.14	12.31 ± 7.12
U2	6.72 ± 4.21	7.79 ± 4.86	**6.16 ± 2.67**	7.72 ± 5.56	6.89 ± 4.51	7.09 ± 4.80
U3	**4.34 ± 2.87**	5.06 ± 3.41	4.43 ± 2.07	5.81 ± 3.80	4.71 ± 3.18	4.56 ± 3.09
U4	5.51 ± 5.01	**4.29 ± 2.18**	6.03 ± 2.27	4.55 ± 2.31	4.39 ± 2.26	4.41 ± 2.29
U5	10.87 ± 4.77	**10.50 ± 4.35**	13.94 ± 4.44	12.18 ± 4.97	10.50 ± 4.36	11.17 ± 4.41
U6	17.21 ± 5.50	5.28 ± 2.17	7.40 ± 3.40	5.41 ± 2.23	**5.28 ± 2.22**	5.37 ± 2.26
U7	14.69 ± 6.40	7.68 ± 3.41	10.71 ± 5.24	8.01 ± 3.57	**7.66 ± 3.46**	8.04 ± 3.91
FDI16 Dataset						
U6	26.96 ± 37.56 ^f^	4.11 ± 3.12 ^b^	6.39 ± 3.89 ^e^	4.21 ± 3.22 ^c^	**4.09 ± 3.27 ^a^**	4.22 ± 4.03 ^d^

Methods sharing the same superscript letter are not significantly different (Wilcoxon signed-rank, Bonferroni-corrected *p* > 0.05). Letter groups are computed independently for each dataset.

## Data Availability

The alignment code and dataset used in this study are available at https://github.com/superbijk/ToothGenNet (accessed on 28 February 2026).

## Supplementary Materials

The following supporting information can be downloaded at: https://www.mdpi.com/article/10.3390/jimaging12060243/s1, Figure S1: Training convergence for four trainable methods (PointNet, PointNetLK, rPointNet, gPCA-rPointNet) over 50,000 training steps and three non-trainable baselines (ICP, PCA, gPCA, shown as dashed lines). (**Top-left**) SE(3) composition training loss. (**Top-right**) Validation geodesic rotation error (degrees). (**Bottom-left**) Validation CD Target (×10^−3^). (**Bottom-right**) Validation CD Template (×10^−3^); Figure S2: Per-axis rotation error distributions and total angular deviation by method on the training validation set (*n* = 1805). (**Top-left**) Mesio-distal (X-axis) rotation component. (**Top-right**) Bucco-lingual (Y-axis) rotation component. (**Bottom-left**) Occluso-gingival (Z-axis) rotation component. (**Bottom-right**) Geodesic angular deviation. Density is capped at 0.20 for clarity; Figure S3: Alignment quality by CD Template bin for U6 first molars on the training validation set (*n* = 1805) across six methods (PointNetLK excluded). CD Template bins (×10^−3^): <5, 5–10, 10–20, >20; Figure S4: Alignment quality by CD Template bin for external FDI16 first molars (*n* = 1465) across six methods (PointNetLK excluded). CD Template bins (×10^−3^): <5, 5–10, 10–20, >20; Figure S5: Interactive web application for the canonicalization pipeline, shown on an example upper left second premolar (FDI 25) aligned by gPCA-rPointNet. The aligned mesh (red) is overlaid on the canonical template (blue). The user selects a dataset sample or uploaded mesh, and the side panel reports per-stage vertex statistics and CD Template; Table S1: Mean rotation error (degrees), translation distance (mm), and Chamfer Distance (×10^−3^) with bootstrap 95% confidence intervals, by method and tooth class. Values are mean (95% CI lower, upper).

## Abbreviations

The following abbreviations are used in this manuscript:

CD	Chamfer distance
FDI	FDI World Dental Federation two-digit tooth numbering system
ICP	Iterative closest point
PCA	Principal component analysis
gPCA	PCA with PointNet-guided candidate selection
PointNet	Permutation-invariant point cloud encoder by Qi et al.
PointNetLK	PointNet with Lucas–Kanade alignment
rPointNet	Residual PointNet correction
gPCA-rPointNet	gPCA with residual PointNet refinement
SE(3)	Special Euclidean group (3D rigid transforms)
SO(3)	Special orthogonal group (3D rotations)
