# Supplementary material for: Neural Residual Correction for 3D Tooth Point Cloud Canonicalization
Source: J Imaging. 2026 May 29;12(6):243. doi: 10.3390/jimaging12060243 (PMC13301075; doi:10.3390/jimaging12060243)
Supplement: Supplementary file 1 [file jimaging-12-00243-s001.zip › jimaging-4205808-supplementary.pdf]

Supplementary Materials

Supplementary Table S1. Mean rotation error (degrees), translation distance (mm), and Chamfer Distance (× 10^{-3}) with bootstrap 95% confidence intervals, by method and tooth class. Values are mean (95% CI lower, upper).

Tooth Class	ICP	PCA	PointNet	rPointNet	gPCA	gPCA-rPointNet
**Training Dataset - Geodesic rotation error (degrees)**						
All	20.42 (18.90-21.97)^{d}	32.40 (29.40-35.39)^{d}	13.50 (12.60-14.43)^{c}	11.40 (9.71-13.20)^{b}	5.92 (5.07-6.84)^{b}	**3.33 (2.71-4.02)**^{a}
U1	3.78 (2.25-5.65)	45.05 (36.76-53.76)	8.30 (7.67-9.02)	5.69 (3.32-8.67)	5.71 (4.00-7.70)	**2.00 (1.59-2.48)**
U2	**3.07 (2.58-3.80)**	63.12 (54.11-72.13)	12.00 (10.14-14.36)	9.57 (6.65-12.98)	12.54 (9.18-16.21)	5.08 (3.16-7.50)
U3	3.48 (2.47-5.20)	21.72 (15.82-28.21)	9.65 (8.83-10.69)	9.24 (5.55-13.59)	6.44 (5.07-8.21)	**3.21 (2.19-4.89)**
U4	9.31 (6.80-12.25)	3.88 (1.93-6.47)	10.38 (9.32-11.79)	2.41 (1.07-4.37)	**1.57 (1.44-1.71)**	1.85 (1.15-3.14)
U5	14.58 (10.99-18.64)	46.51 (38.01-55.33)	14.85 (12.45-17.60)	17.21 (11.93-23.11)	7.29 (5.38-9.80)	**6.21 (3.59-9.42)**
U6	62.58 (59.97-65.14)	18.03 (12.16-24.05)	16.51 (14.14-19.14)	18.26 (12.38-24.24)	2.66 (0.90-5.00)	**1.77 (1.11-2.99)**
U7	60.63 (54.30-67.06)	22.25 (13.32-31.52)	30.17 (24.21-36.90)	22.49 (13.64-31.69)	4.92 (1.58-9.33)	**3.50 (1.99-6.12)**
**Training Dataset - Translation (mm)**						
All	0.29 (0.27-0.31)^{c}	0.30 (0.29-0.31)^{d}	0.53 (0.52-0.54)^{f}	0.35 (0.33-0.37)^{e}	0.19 (0.18-0.20)^{b}	**0.15 (0.14-0.16)**^{a}
U1	0.31 (0.23-0.40)	0.37 (0.33-0.41)	0.66 (0.63-0.68)	0.64 (0.57-0.72)	0.24 (0.20-0.28)	**0.17 (0.14-0.21)**
U2	**0.13 (0.11-0.14)**	0.40 (0.37-0.44)	0.54 (0.51-0.58)	0.47 (0.42-0.53)	0.26 (0.23-0.30)	0.18 (0.15-0.21)
U3	**0.13 (0.12-0.14)**	0.32 (0.29-0.35)	0.60 (0.57-0.64)	0.45 (0.41-0.50)	0.30 (0.27-0.34)	0.21 (0.18-0.23)
U4	0.22 (0.18-0.27)	0.28 (0.25-0.30)	0.44 (0.42-0.47)	0.22 (0.20-0.24)	**0.10 (0.09-0.10)**	0.10 (0.09-0.11)
U5	0.27 (0.22-0.31)	0.31 (0.28-0.34)	0.44 (0.41-0.47)	0.24 (0.21-0.27)	0.17 (0.15-0.18)	**0.13 (0.11-0.14)**
U6	0.56 (0.52-0.60)	0.20 (0.16-0.24)	0.47 (0.45-0.49)	0.20 (0.16-0.24)	0.11 (0.10-0.13)	**0.11 (0.10-0.12)**
U7	0.46 (0.41-0.51)	0.16 (0.12-0.19)	0.58 (0.54-0.63)	0.17 (0.14-0.20)	**0.11 (0.09-0.13)**	0.16 (0.12-0.20)
**Training Dataset - CD Target (× 10^{-3})**						
All	3.05 (2.76-3.38)^{c}	1.60 (1.49-1.71)^{c}	2.66 (2.53-2.80)^{e}	1.71 (1.55-1.89)^{d}	0.74 (0.63-0.87)^{b}	**0.62 (0.52-0.74)**^{a}
U1	3.63 (2.19-5.29)	2.86 (2.45-3.31)	2.81 (2.55-3.14)	3.02 (2.35-3.86)	1.34 (0.78-2.02)	**1.07 (0.52-1.79)**
U2	**0.27 (0.24-0.31)**	2.47 (2.11-2.86)	1.88 (1.68-2.13)	2.74 (2.19-3.38)	1.16 (0.81-1.57)	0.76 (0.55-1.00)
U3	**0.30 (0.28-0.33)**	1.47 (1.27-1.69)	2.23 (2.03-2.44)	2.02 (1.76-2.31)	0.85 (0.69-1.07)	0.78 (0.61-0.99)
U4	1.53 (1.09-2.05)	1.06 (0.95-1.17)	1.80 (1.62-2.00)	1.01 (0.92-1.12)	**0.27 (0.27-0.28)**	0.28 (0.27-0.31)
U5	1.32 (0.92-1.75)	1.11 (0.97-1.27)	2.43 (2.01-2.94)	0.95 (0.82-1.09)	0.47 (0.37-0.60)	**0.34 (0.30-0.41)**
U6	8.94 (8.52-9.34)	0.87 (0.69-1.08)	3.33 (3.02-3.67)	0.87 (0.70-1.07)	0.48 (0.43-0.56)	**0.47 (0.44-0.51)**
U7	5.95 (5.36-6.54)	1.03 (0.77-1.31)	5.37 (4.66-6.19)	1.05 (0.79-1.32)	**0.49 (0.39-0.64)**	0.62 (0.47-0.83)
**Training Dataset - CD Template (× 10^{-3})**						
All	10.23 (9.90-10.56)^{d}	7.63 (7.40-7.86)^{c}	8.50 (8.28-8.71)^{e}	8.24 (7.99-8.49)^{d}	**7.39 (7.16-7.61)**^{a}	7.57 (7.33-7.81)^{b}
U1	13.10 (12.16-14.12)	12.59 (11.96-13.26)	**11.57 (11.17-12.01)**	13.76 (13.06-14.50)	12.19 (11.50-12.93)	12.31 (11.53-13.16)
U2	6.72 (6.25-7.21)	7.79 (7.24-8.35)	**6.16 (5.87-6.47)**	7.72 (7.11-8.37)	6.89 (6.39-7.42)	7.09 (6.56-7.65)
U3	**4.34 (3.99-4.72)**	5.06 (4.63-5.50)	4.43 (4.17-4.71)	5.81 (5.33-6.30)	4.71 (4.31-5.13)	4.56 (4.17-4.96)
U4	5.51 (4.95-6.10)	**4.29 (4.04-4.54)**	6.03 (5.78-6.30)	4.55 (4.28-4.82)	4.39 (4.13-4.65)	4.41 (4.15-4.68)
U5	10.87 (10.30-11.45)	**10.50 (9.97-11.01)**	13.94 (13.42-14.48)	12.18 (11.58-12.78)	10.50 (9.98-11.03)	11.17 (10.64-11.70)
U6	17.21 (16.58-17.81)	5.28 (5.04-5.53)	7.40 (7.02-7.79)	5.41 (5.16-5.67)	**5.28 (5.03-5.53)**	5.37 (5.11-5.63)
U7	14.69 (13.64-15.72)	7.68 (7.13-8.25)	10.71 (9.91-11.60)	8.01 (7.44-8.61)	**7.66 (7.10-8.23)**	8.04 (7.42-8.69)
**FDI16 Dataset - CD Template (× 10^{-3})**						
U6	26.96 (25.04-28.95)^{f}	4.11 (3.95-4.27)^{b}	6.39 (6.19-6.59)^{e}	4.21 (4.05-4.38)^{c}	**4.09 (3.93-4.26)**^{a}	4.22 (4.02-4.44)^{d}

Methods sharing the same superscript letter in the All row are not significantly different (Wilcoxon signed-rank, Bonferroni-corrected $p > 0.05$).

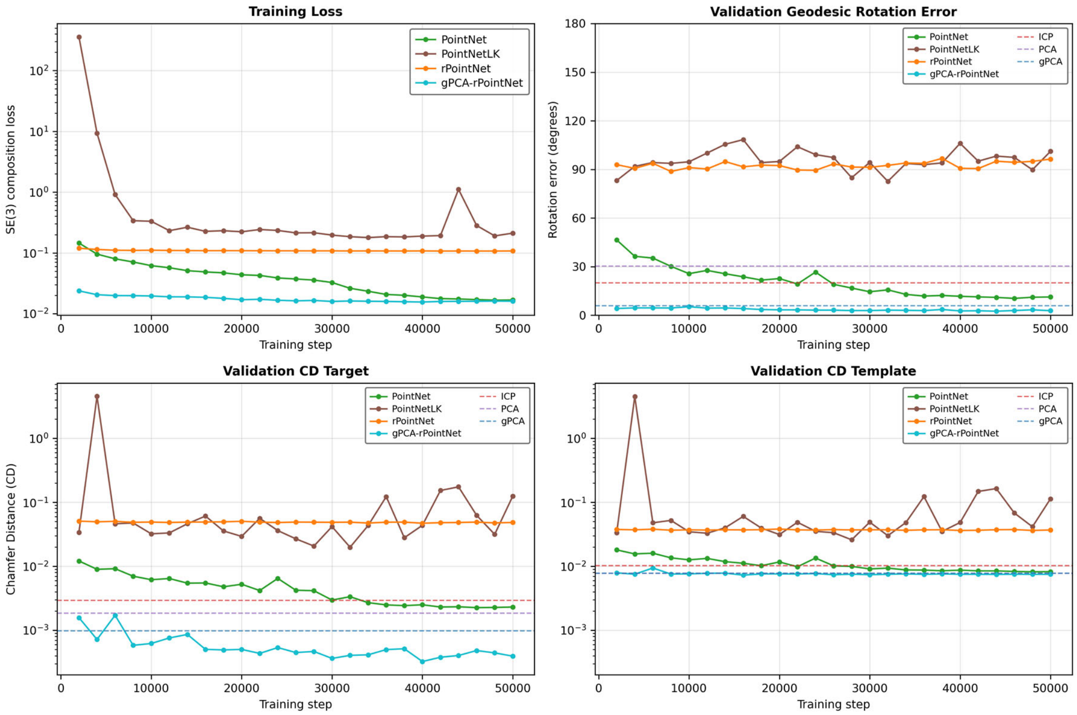

Supplementary Figure S1. Training convergence for four trainable methods (PointNet, PointNetLK, rPointNet, gPCA-rPointNet) over 50,000 training steps and three non-trainable baselines (ICP, PCA, gPCA, shown as dashed lines). (**Top-left**) SE(3) composition training loss. (**Top-right**) Validation geodesic rotation error (degrees). (**Bottom-left**) Validation CD Target (× 10^{-3}). (**Bottom-right**) Validation CD Template (× 10^{-3}).

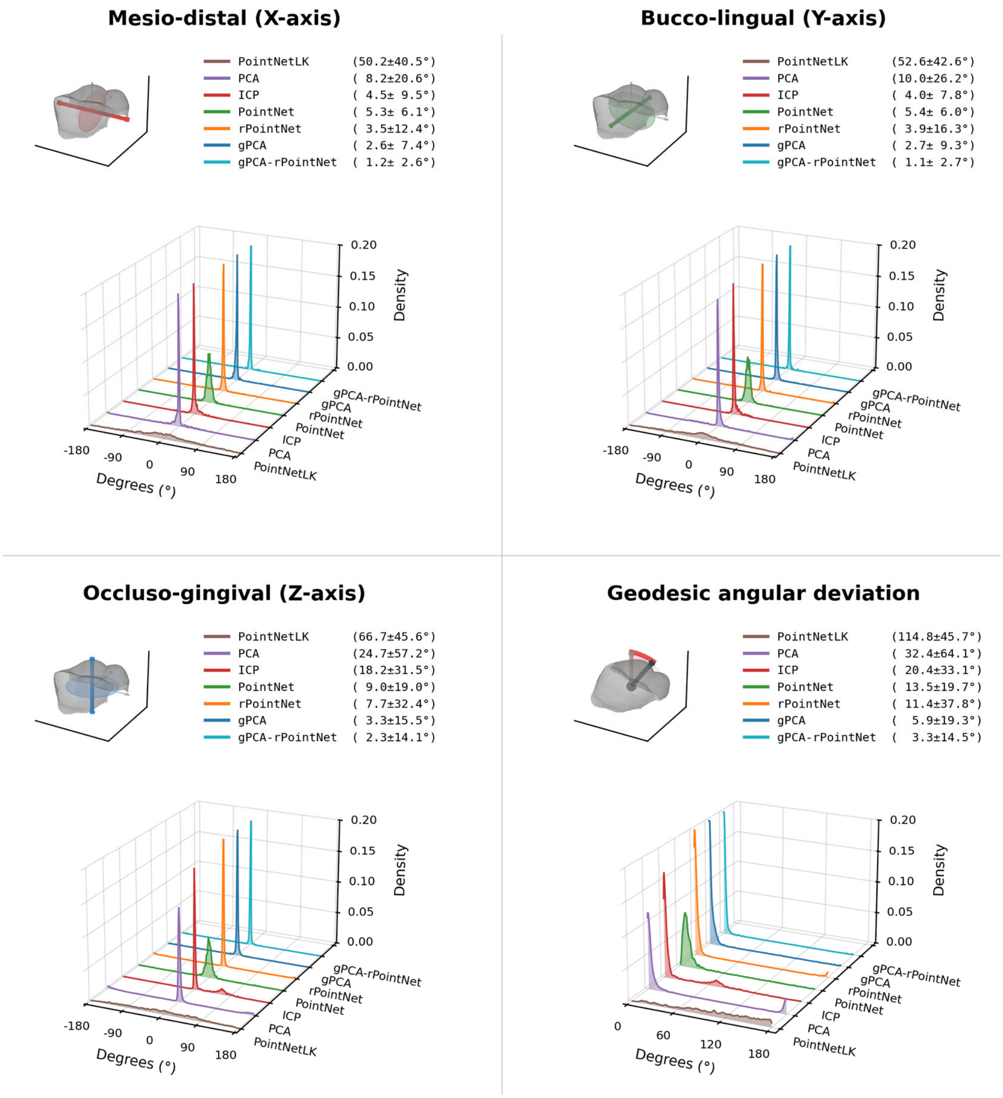

Supplementary Figure S2. Per-axis rotation error distributions and total angular deviation by method on the training validation set ($n = 1805$). (**Top-left**) Mesio-distal (X-axis) rotation component. (**Top-right**) Bucco-lingual (Y-axis) rotation component. (**Bottom-left**) Occluso-gingival (Z-axis) rotation component. (**Bottom-right**) Geodesic angular deviation. Density is capped at 0.20 for clarity.

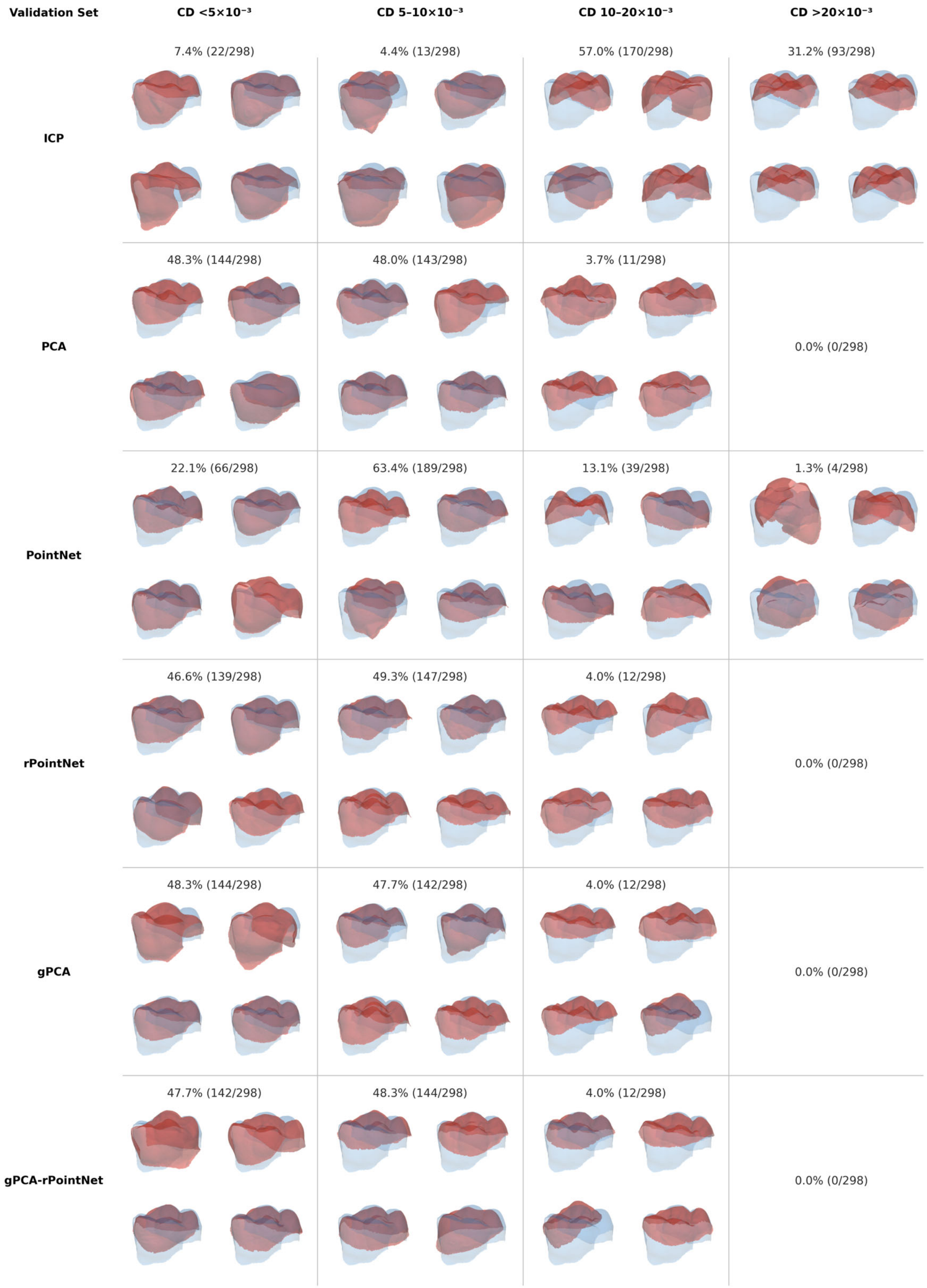

Supplementary Figure S3. Alignment quality by CD Template bin for U6 first molars on the training validation set (n = 1805) across six methods (PointNetLK excluded). CD Template bins (× 10^{-3}): <5, 5–10, 10–20, >20.

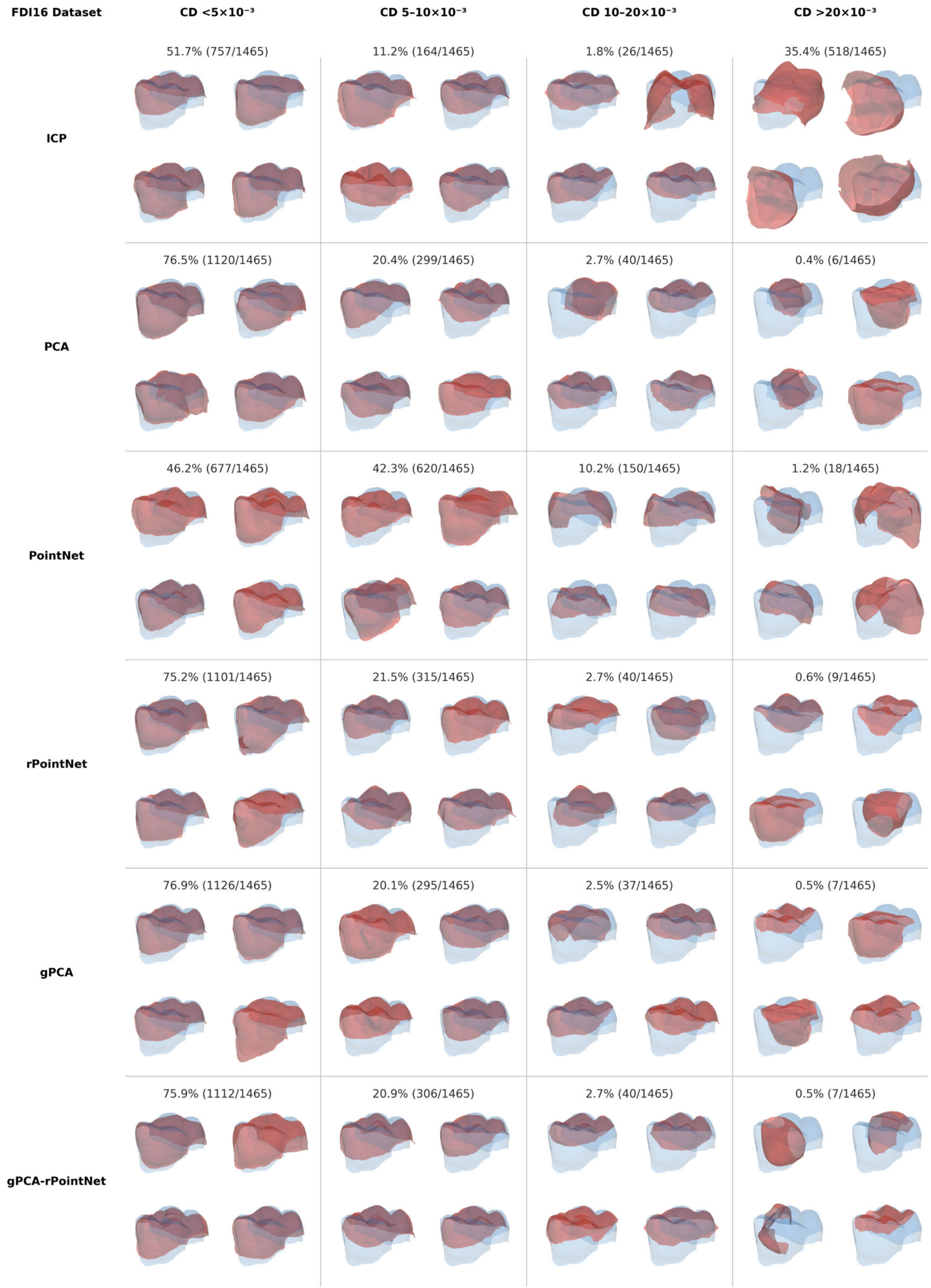

Supplementary Figure S4. Alignment quality by CD Template bin for external FDI16 first molars ($n = 1465$) across six methods (PointNetLK excluded). CD Template bins ($\times 10^{-3}$): <5, 5–10, 10–20, >20.

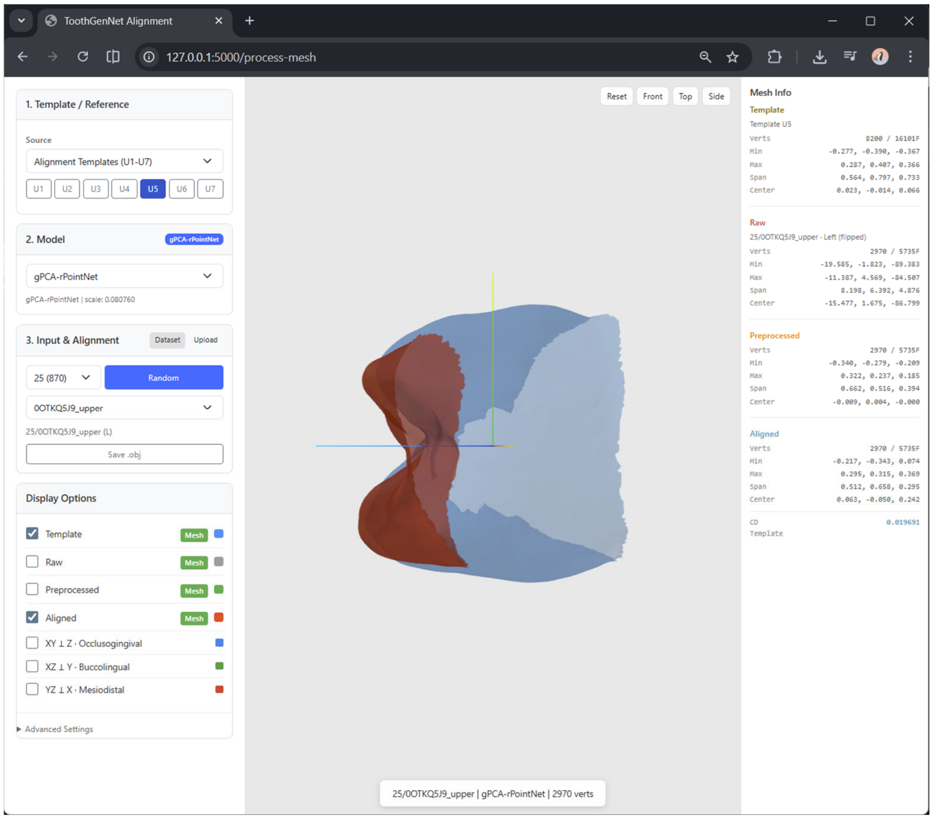

Supplementary Figure S5. Interactive web application for the canonicalization pipeline, shown on an example upper left second premolar (FDI 25) aligned by gPCA-rPointNet. The aligned mesh (red) is overlaid on the canonical template (blue). The user selects a dataset sample or uploaded mesh, and the side panel reports per-stage vertex statistics and CD Template.
